# Flexural Strength Properties of Five Different Monolithic Computer-Aided Design/Computer-Aided Manufacturing Ceramic Materials: An In Vitro Study

**DOI:** 10.7759/cureus.36958

**Published:** 2023-03-31

**Authors:** Esraa A Attar, Ayman Aldharrab, Reem Ajaj

**Affiliations:** 1 Department of Oral and Maxillofacial Prosthodontics, King Abdulaziz University, Jeddah, SAU; 2 Department of Restorative Dentistry, King Abdulaziz University, Jeddah, SAU

**Keywords:** lithium disilicate, lucite ceramics, translucent zirconia, weibull modulus, flexural strength

## Abstract

Objective

The purpose of this in vitro study is to compare the flexural strength and Weibull modulus of 5 different monolithic computer-aided design/computer-aided manufacturing (CAD/CAM) ceramics.

Methods

A total of 50 specimens were fabricated, 10 from each of the following materials: lithium disilicate-based ceramic (IPS e.max CAD), zirconia -reinforced lithium-silicate ceramic (Vita Suprinity), leucite-based glass ceramic (IPS Empress CAD), and two zirconia-based ceramics (Zenostar and CopraSmile). The specimens were 4 mm wide, 2 mm thick, and 16 mm long. Flexural strength test was executed using a universal testing machine (Model 5980, Instron Industrial Products, Norwood, MA, USA). The two-parameter Weibull distribution function was used to analyze the variability of flexural strength values. Statistical analysis was performed on SPSS Version 23 (IBM Corp., Armonk, NY, USA) using one-way analysis of variance (ANOVA) and post-hoc Tukey’s test.

Results

Suprinity had the highest Weibull modulus value, while Empress CAD displayed the lowest value. One-way ANOVA showed significant difference in the flexural strength between the different materials tested (p*<*0.05)*. *Post-hoc analysis revealed significant differences among all the test groups in terms of flexural strength. Zenostar presented the highest mean flexural strength value (1033.90 MPa), while Empress CAD had the lowest value.

Conclusion

High-translucency zirconia had superior flexural properties than translucent zirconia, lithium disilicate ceramics, zirconia-reinforced lithium silicate ceramics, and leucite-based glass ceramics.

## Introduction

In modern dentistry, the use of computer-aided design/computer-aided manufacturing (CAD/CAM) ceramics has greatly increased. In search of the ultimate esthetic ceramic material, various CAD/ CAM ceramics have been introduced with superior mechanical and esthetic characteristics [1-4]. Ceramics can be classified into three general categories according to their microstructure [5-7]:

1. Predominantly glass ceramics, which have a high-glass content, with small amounts of filler particles to improve the optical properties of the materials. Lucite-reinforced glass ceramics (40-50% Lucite) are examples of this kind of ceramics.

2. Particle-filled glass ceramics, which have a low-glass content, with added filler particles to improve the mechanical properties. These fillers usually are crystalline or of high-melting glasses that are dissolved during etching to create micromechanical retentive features enabling bonding. Lithium disilicate (LD) ceramics (70% LD) belong to this category.

3. Polycrystalline ceramics, which contain no glass phase, and all the crystals are arranged in a dense matrix of crystalline arrays, which is highly resistant to crack progression, such as alumina and zirconia ceramics.

All the previously mentioned categories have been produced using CAD/CAM technology, with superior homogeneity and minimal flaws. The advancements in CAD/CAM technology have resulted in the development of high-strength yet aesthetic ceramic restorations with superior biomechanical properties [8,9].

Lucite-reinforced glass ceramics consist of a silicate glass matrix reinforced with lucite crystals (SiO_2_-Al_2_O_3_-K_2_). The greatest advantage of this class of ceramics is their exceptional esthetics. They are manufactured either by heat pressing using the lost wax technique or by milling. The ingots (e.g., IPS Empress Esthetic) and blocks (e.g., IPS Empress CAD) are supplied in different shades and translucencies. These restorations are supplied in a fully crystallized stage and can be stained, characterized, polished, and glazed [10,11].

LD restorations are low-glass-content, particle-filled glass ceramics that are produced either by heat-pressing or milling. The final resertoration produced contains 70% LD (Li2Si2O5) crystals that contribute mainly to reducing crack propagation and to the superior mechanical properties of LD materials. LD used in the heat pressing technique is provided in the form of ingots pressed using the lost wax technique, e.g., IPS e.max Press. CAD/CAM blocks, e.g., IPS e.max CAD are manufactured in a partially crystallized stage (Li_2_SiO_3_) necessary for the milling procedure. This requires firing after milling to reach final crystallization by passing through nucleation and crystal growth [6,10-12]. This firing improves the mechanical and optical properties of the material, and Li_2_SiO_3_ crystals transform into Li_2_Si_2_O_5_ crystals. The final translucency of LD-based ceramics is obtained when the lithium metasilicate is dissolved and LD is crystallized [13,14]. This leads to an expansion in the number of crystals by 70% in volume, and flexural strength increases to 360-400 MPa [8].

To improve the mechanical properties of LD ceramics, the manufacturers introduced zirconia-reinforced LD (ZLD) glass-ceramic materials, e.g., Vita Suprinity. It contains dual microstructure in the glass matrix: LD crystals and 10% zirconium oxide (ZrO_2_). The CAD/CAM blocks are manufactured in a precrystallized stage, and after firing, they reach their fully crystallized form [15,16].

CAD/CAM zirconia-based ceramics are manufactured in pre-sintered blocks of Yttria-stabilized polycrystalline tetragonal zirconia (Y-TZP). After milling, then sintering under high temperature, transformation toughening takes place. It is responsible for the transformation from tetragonal to monoclinic ZrO_2_, therefore, increasing the flexural strength and resistance to crack propagation. The major drawback of zirconia-based restorations is their low translucency, which led to the introduction of high-translucency Y-TZP to improve esthetics. The manufacturer increased the content of yttrium Oxide (Y_2_O_3_), partially replacing tetragonal zirconia with cubic zirconia, which improved translucency but subsequently reduced the flexural strength and fracture toughness [11,17-19].

The mechanical properties of ceramics have been comprehensively investigated in previous studies. The flexural strength of dental ceramics is measured by multiple tests, such as three-point flexural test, four-point flexural test, or biaxial flexural test [20-22]. Flexural strength and Weibull modulus are used to evaluate the stiffness of the material through the resistance to failure from bending. The three-point flexural strength test subjects the specimen to tensile, compressive, and shear stress [10].

The purpose of this in vitro study is to compare the flexural strength of five different monolithic CAD/CAM ceramic materials: LD-based ceramic (IPS-e.max CAD), ZLD ceramic (VITA Suprinity), leucite-based glass ceramic (IPS Empress CAD), and two zirconia-based ceramics (translucent zirconia Zenostar T, and high-translucency zirconia CopraSmile).

## Materials and methods

The study has investigated a total of five CAD/CAM ceramic materials (n=50). Five groups (n=10) were included. The materials tested and their composition and manufacturer are presented in (Table 1).

**Table 1:** The tested materials, composition, and manufacturer

Material	Composition	Manufacturer
IPS-emax CAD	Lithium disilicate-based ceramic containing 70% Li_2_Si_2_O_5 _crystals	Ivoclar Vivadent, Schaan, Liechtenstein
VITA Suprinity	Zirconia-reinforced lithium silicate ceramic containing Li_2_Si_2_O_5 _crystals and 10% ZrO_2_	Vita Zahnfabric, Bad Säckingen, Germany
IPS Empress CAD	Leucite-based glass ceramic based on SiO_2_-Al_2_O_3_-K_2_O material system	Ivoclar Vivadent, Schaan, Liechtenstein
Zenostar	High translucency zirconia-based ceramic composed of Y-TZP, ZrO_2_, HFO_2_, Y_2_O_3_	Ivoclar Vivadent, Schaan, Liechtenstein
CopraSmile	Translucent zirconia-based ceramic composed of Y-TZP, ZrO_2_, HFO_2_, Y_2_O_3_	Whitepeaks Dental Solutions GmbH & Co. KG, Essen, Germany

Specimen preparation

All specimens were fabricated according to the manufacturer’s instructions. Bar-shaped specimens were CAD/CAM milled using CEREC inLab MC XL milling unit (Sirona Dental Systems Gmbh, Bensheim, Germany) according to the ISO 6872:2015 standard, with the following dimensions: 4 mm in width, 2 mm in thickness, and 16 mm in length.

Blocks of IPS Empress CAD were milled in a fully sintered stage, IPS e.max CAD and Vita Suprinity were milled in a pre-crystallized stage, and CopraSmile and Zenostar were milled in a partially sintered stage. The furnace used for sintering and crystallization of the samples was Programat S1 (Ivoclar Vivadent, Schaan, Liechtenstein). Zenostar samples were sintered using for a duration of 9 hours and 50 minutes, and CopraSmile samples were sintered for a duration of 8 hours. IPS-e.max CAD samples were crystallized for 30 minutes, and Vita Suprinity samples were crystallized for 26 minutes. The specimen edge was grounded lengthwise along the long axis of the specimen to a 45-degree chamfer so that chipping was minimized. The edge of the chamfer was grounded prior to the final sintering for the zirconia specimens.

Flexural strength testing

Three-point flexural strength testing was conducted according to ISO 6872:2015 standard in a universal testing machine (Model 5980; Instron Industrial Products. Norwood, MA, USA). The specimens were positioned on the support rollers with their centers 12 mm apart. Load was applied at the midpoint by means of a third roller at a crosshead speed of 0.5 mm/min until failure. Failure loads were recorded, and mean values of flexural strength were calculated using the following equation:

*σ*=3*pl*/2*wb*2

where *p* is the breaking load in Newtons, *l *is a center-to-center distance between the support rollers in millimeters, *w* is the width of the specimens in millimeters, and *b* is the thickness of the specimens in millimeters.

Statistical analysis was performed using SPSS Version 23 (IBM Corp., Armonk, NY, USA). Descriptive statistics in terms of means of flexural strength and standard deviations were calculated for each group. One-way analysis of variance (ANOVA) was performed to compare the difference in flexural strength between the five ceramic groups. Tukey’s honest significant difference post-hoc test was used to compare which specific pairs of means were significantly different. Statistical tests were conducted at a significance level of p<0.05.

The two-parameter Weibull distribution function was used to analyze the variability of flexural strength values. The Weibull modulus was calculated using the following equation

*pf*(*σ*)=1-exp [-(*σ*/*σ*0)*m*]

where *pf*(*σ*) is the probability of failure, *σ* is the fracture strength, σ0 is the characteristic strength, and *m* is the Weibull modulus.

Then, the probability of failure based on its ranking was calculated using the following equation

*pf*=*i*-0.5/*n*

where *i* is ranking and *n* is the number of specimens.

## Results

Means, standard deviations, and Weibull modulus for flexural strength for the five tested groups are shown in Table 2.

**Table 2:** Analysis of the measured flexural strength

Material	Mean flexure strength (Mpa)	SD	Weibull modulus (m)
IPS e.max CAD	372.68	24.10	14.93
IPS Empress CAD	137.37	10.97	12.26
VITA Suprinity	428.48	12.39	34.48
Zenostar	1033.90	58.78	16.64
CopraSmile	621.79	30.09	20.32

**Figure 1:**
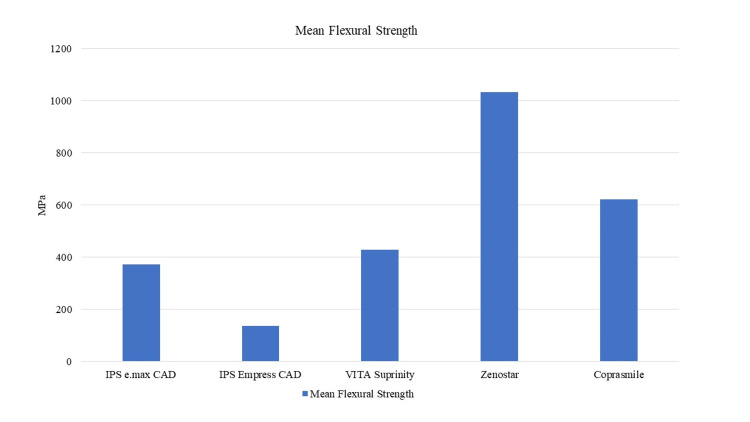
Mean flexural strength for the five ceramic groups

Figure 1 illustrates the mean flexural strength for the five different groups. Zenostar group presented the highest mean flexural strength value (1033.90 MPa), while Empress CAD had the lowest value (137.37 MPa).

**Figure 2:**
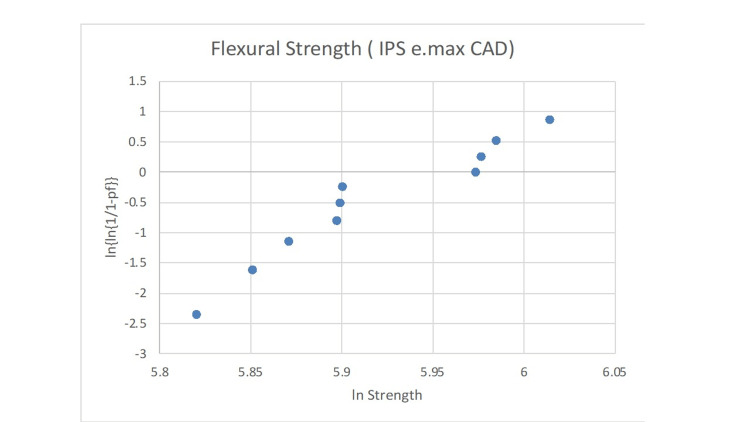
Failure probability curve of the Weibull modulus for the IPS e.max CAD group

**Figure 3:**
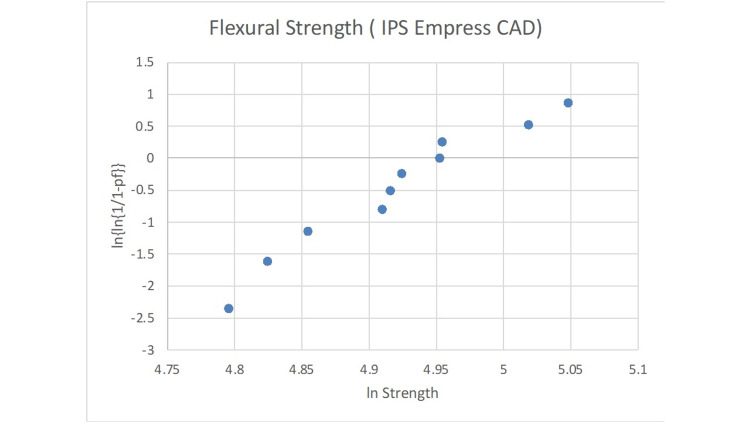
Failure probability curve of the Weibull modulus for the IPS Empress CAD group

**Figure 4:**
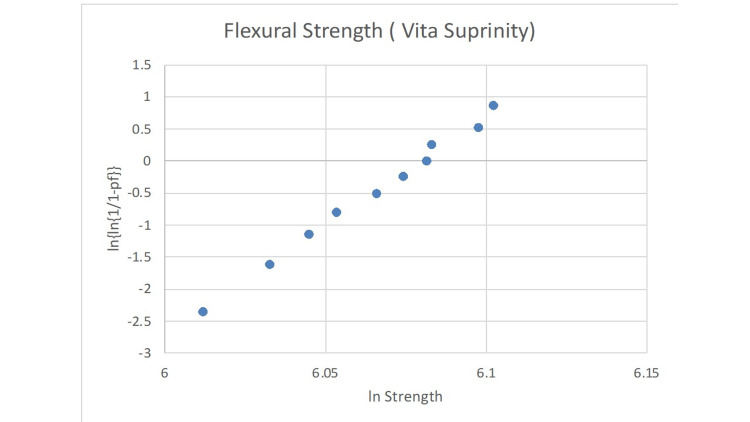
Failure probability curve of the Weibull modulus for the Vita Suprinity group

**Figure 5:**
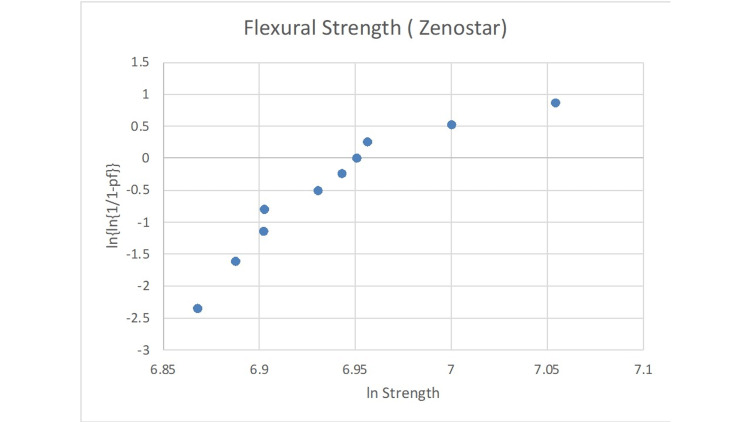
Failure probability curve of the Weibull modulus for the Zenostar group

**Figure 6:**
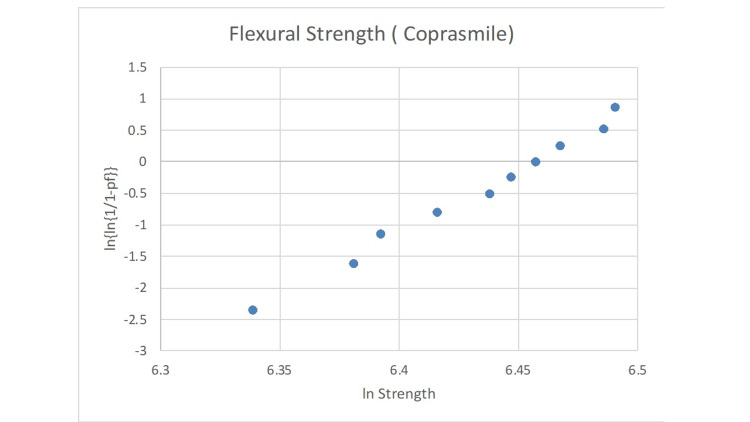
Failure probability curve of the Weibull modulus for the CopraSmile group

The statistical analysis of the Weibull modulus is presented in Figures 2-6. The Suprinity group had the highest Weibull modulus value, while Empress CAD displayed the lowest value.

One-way ANOVA for the mean flexural strength of the five ceramics is presented in Table 3. The results show a significant difference in the flexural strength between the different materials tested (p<0.05).

**Table 3:** One-way ANOVA of the mean flexural strength values of the tested ceramic materials

	Sum of squares	df	Mean square	F	Sig.
Between groups	4,505,574.752	4	1,126,393.688	1,080.018	0.000
Within groups	46,932.294	45	1,042.940		
Total	4,552,507.046	49			

Table 4 shows the results of the post-hoc analysis (Tukey’s test) to indicate significant differences among all the test groups in terms of flexural strength. From the results, it is clear that all the specimens were significantly differentiated based on their mean flexural strength.

**Table 4:** Post-hoc analysis (Tukey’s test) of the flexural strength for the tested ceramic materials

I	J	Mean difference (I-J)	Std. error	Sig.	95% confidence interval	
					Lower bound	Upper bound
e.max	Empress	235.30800^*^	14.44258	0.000	194.2701	276.3459
	Suprinity	-57.90700^*^	14.44258	0.002	-98.9449	-16.8691
	Zenostar	-661.22300^*^	14.44258	0.000	-702.2609	-620.1851
	CopraSmile	-249.11300^*^	14.44258	0.000	-290.1509	-208.0751
Empress	e.max	-235.30800^*^	14.44258	0.000	-276.3459	-194.2701
	Suprinity	-293.21500^*^	14.44258	0.000	-334.2529	-252.1771
	Zenostar	-896.53100^*^	14.44258	0.000	-937.5689	-855.4931
	CopraSmile	-484.42100^*^	14.44258	0.000	-525.4589	-443.3831
Suprinity	e.max	57.90700^*^	14.44258	0.002	16.8691	98.9449
	Empress	293.21500^*^	14.44258	0.000	252.1771	334.2529
	Zenostar	-603.31600^*^	14.44258	0.000	-644.3539	-562.2781
	CopraSmile	-191.20600^*^	14.44258	0.000	-232.2439	-150.1681
Zenostar	e.max	661.22300^*^	14.44258	0.000	620.1851	702.2609
	Empress	896.53100^*^	14.44258	0.000	855.4931	937.5689
	Suprinity	603.31600^*^	14.44258	0.000	562.2781	644.3539
	CopraSmile	412.11000^*^	14.44258	0.000	371.0721	453.1479
CopraSmile	e.max	249.11300^*^	14.44258	0.000	208.0751	290.1509
	Empress	484.42100^*^	14.44258	0.000	443.3831	525.4589
	Suprinity	191.20600^*^	14.44258	0.000	150.1681	232.2439
	Zenostar	-412.11000^*^	14.44258	0.000	-453.1479	-371.0721
*The mean difference is significant at the 0.05 level.						

## Discussion

Manufacturers and material scientists are constantly seeking the most esthetically pleasing and durable ceramic material to meet the growing demand for esthetic dental ceramics. However, these ceramics are brittle in nature and subject to failure under the challenges in the oral environment. Therefore, this study compared the flexural strength and Weibull modulus of five of the currently machined ceramics used for the construction of single crowns, and minimal and non-prep veneers. The ceramics investigated were IPS-e.max CAD, VITA Suprinity, IPS Empress CAD, Zenostar, and CopraSmile.

The results of the flexural strength testing for these materials displayed that Zenostar had the highest flexural strength value (1,033.90 MPa). This can be explained as Zenostar material is made of densely sintered ZrO_2_ with some additives to add translucency to the material. Previous studies reported similar values for the flexural strength of Zenostar to be between 900 and 954 MPa [23]. CopraSmile had the second highest flexural strength value (621.79 MPa), which corresponds to the reduction in zirconium oxide contents to improve translucency. This value was higher than that reported previously by Nassary et al. for the three-point flexural strength test of CopraSmile, which was 467 MPa [24]. It has been reported that highly translucent ceramics have lower flexural strengths in comparison to those with lower translucency [25]. They were followed by Vita Suprinity (428.48 MPa) and then IPS e.max CAD (372.68 MPa). Both materials are LD-based ceramics in which Vita Suprinity is reinforced with 10% ZrO_2_ to increase strength. Our results are comparable to previous studies where the authors reported values of the three-point flexural strength test of IPS-e.max CAD between 271.6 and 440 MPa [26-30]. As for crystalized Vita Suprinity, the three-point flexural strength test has been reported to be between 420 and 494.5 MPa [16].

IPS Empress CAD has the lowest flexural strength values among the other tested materials (137.37 MPa); the material is leucite-reinforced glass ceramics, which was reported by other studies to have lower mechanical properties than LD-glass ceramics. Previous studies reported flexural strength values between 146.9 and 151 MPa for IPS Empress CAD [20-22,26,27].

Regarding LD-based ceramics, previous studies reported results similar to this investigation. They reported that ZLD ceramics had higher flexural strength values than LD-based ceramics. This can be attributed to the presence of interlocking crystals in the glassy matrix. ZLD ceramics have a lower percentage of the crystalline phase than LD ceramics, but instead, they were replaced by reinforcing ZrO_2_ particles in the glassy phase. This might have strengthened the material through resistance to crack propagation. De Mendonca et al. compared the mechanical properties of four CAD-CAM materials used in the fabrication of monolithic restorations: LD, ZLD, hybrid high-performance polymer composite resin (HPP), and hybrid polymer-infiltrated ceramic network material (PINC). Results of flexural strength testing showed that LD had the highest flexural strength values of 289 MPa followed by ZLD (230 MPa), HPP, and then PINC. ZLS had the highest flexural modulus and hardness [15]. Another study by Elsaka and Elnaghy compared the mechanical properties of two CAD-CAM ceramics: IPS e.max CAD and Vita Suprinity. Vita Suprinity displayed superior mechanical properties and higher values in terms of flexural strength, fracture toughness, elastic modulus, and hardness. The flexural strength values reported were 443.63 MPa for Vita Suprinity and 348.33 MPa for IPS e.max CAD [16].

Awada and Nathanson reported flexural strength values of 167 MPa for IPS Empress CAD [10]. Al-Thobity and Al-Salman reported values of 364.6 MPa compared to 137.4 MPa in our study [13].

Results of testing the flexural strength of CopraSmile and Zenostar in this investigation revealed that the higher the translucency of zirconia, the lower the flexural strength. This is attributed to the increase in the volume of Y_2_O_3_, which led to the partial replacement of tetragonal ZrO_2_ by cubic ZrO_2_. Tetragonal ZrO_2_ is responsible for the increase in the strength of the material [17]. Fonzar et al. [12] found statistically significant differences in the flexural strength of different translucencies of IPS e.max CAD. The medium translucency group had higher flexural strength than the high translucency and medium opacity groups (397.5 MPa, 346.2 MPa, and 281.2 MPa, respectively). Our study tested MT IPS e.max CAD only (372.7 MPa), and since translucency was an influential factor on flexural strength, future studies should compare different translucencies of the different materials tested.

In this study, the materials did not undergo any surface treatments after sintering and crystallization. The zirconia-based ceramics underwent sintering, the LD-based ceramics underwent crystallization, and the lucite-based ceramic underwent polishing only. The effects of different surface treatments on the flexural strengths of ceramics have been investigated previously and found to be influential. Lima et al. found significant differences in the flexural strength values between the different surface treatments (e.g., hydrofluoric acid+silane, silane, sandblasting, self-etching ceramic primers) among all the LD ceramics tested, including IPS e.max CAD, Suprinity, and Celtra Duo. The hydrofluoric acid+silane and self-etching ceramic primer groups had higher flexural strength values than the control group with no surface treatment [31]. Moreover, another investigation of the effects of different surface treatments of zirconia on flexural strength has shown that it has an effect on the phase transformation and increased monoclinic phase content of zirconia. The different surface treatments included airborne particle abrasion, grinding, or both. The flexural strength values were highest in the airborne-particle-abraded group [32].

It was found that increasing the surface roughness of zirconia increased its flexural strength, while polishing the surfaces decreased the flexural strength. The authors attributed the increase in flexural strength to the induced compressive stress in the zirconia particles leading to blocking crack propagation [17].

The results of our study were acquired from testing milled bars of the material samples of dimensions and shapes that are not representative of the clinical crowns or ceramic veneers. Studies that simulate clinical applications are needed to compare the materials for more clinically relevant results. Challenges of the oral environment were not taken into account, such as cyclic load, humidity, pH, and temperature variations. To give more indication and stronger consensus to guide in material selection, more tests are needed, such as fracture toughness, which measures the material’s ability to resist crack propagation [33].

## Conclusions

High-translucency zirconia had superior flexural properties than translucent zirconia, LD ceramics, zirconia-reinforced lithium silicate ceramics, and leucite-based glass ceramics. Studies that simulate clinical applications and the oral environment are needed to compare the materials for more clinically relevant results. Further mechanical testing of the materials such as fracture toughness and microhardness needs to be investigated.
